# “The stress can be unbearable, but the good times are like finding gold”: A phase one modelling survey to inform the development of a self-help positive reappraisal coping intervention for caregivers of those with autism spectrum disorder

**DOI:** 10.1371/journal.pone.0264837

**Published:** 2022-03-03

**Authors:** Deborah Lancastle, Joanna Hill, Susan Faulkner, Alecia L. Cousins

**Affiliations:** 1 Faculty of Life Sciences and Education, School of Psychology and Therapeutic Studies, University of South Wales, Pontypridd, United Kingdom; 2 Faculty of Medicine, Health and Life Science, Department of Psychology, Swansea University, Swansea, United Kingdom; University of Wyoming College of Health Sciences, UNITED STATES

## Abstract

Caregivers of individuals with ASD can experience various practical, psychological, and social demands and need effective ways of coping to ameliorate the negative effects of caregiving. Numerous coping strategies are available, but the literature shows that caregivers can still struggle to cope, suggesting that interventions to support coping efforts could be beneficial. The MRC framework advocates the systematic development and evaluation of interventions, and this study was conducted to inform the future development of a self-help Positive Reappraisal Coping Intervention (PRCI) for these caregivers. The aim was to establish whether positive reappraisal coping strategies were used and associated with greater psychological wellbeing, prior to developing such an intervention. **Method.** Caregivers of individuals with ASD (N = 112) responded to items from an existing PRCI (Lancastle, 2006; Lancastle & Boivin, 2008), by writing about aspects of caregiving that reflected the meaning of each item. They also completed questionnaires assessing resilience, caregiving burden, and positive and negative emotions. **Results.** Participants provided significantly more positive responses than negative responses to PRCI items, demonstrating their use of positive reappraisal coping. Thematic analyses showed that positive responses focused on factors such as their loved one’s personality and achievements, the contributions caregivers had made to this person’s progress, the support received, and their own personal development. Positive reappraisal coping was associated with greater resilience, more positive and less negative emotions, and a lesser sense of caregiver burden. **Conclusion.** This modelling study suggests that positive reappraisal strategies were used by caregivers and associated with greater psychological wellbeing. The findings will inform the development of a self-help PRCI for the caregivers of those with ASD. Future studies will systematically evaluate that PRCI to determine the nature of intervention effects and mediators and moderators of effects.

## Introduction

Caregivers of people with Autism Spectrum Disorder (ASD) experience various challenges from caregiving, including greater stress, negative impacts on relationships, financial difficulties, and physical and mental health problems [1,2]. Caregiving parents report less marriage satisfaction and are around twice as likely to become divorced as parents whose children do not have ASD or who have other disabilities [3]. Furthermore, 60% of mothers of a child who had ASD could not continue with their careers, whilst those who remained employed reported reduced working hours and loss of earnings [4]. Psychological impacts on caregivers include loss of autonomy and control, emotional burden, and stress [2,5]. Caregiver stress is moderated by the severity of ASD symptoms, with more severe symptoms predicting greater stress [6–8], more family problems, and elevated pessimism [6], and by the caregiver’s age. Older adults may feel more competent in their caregiving role because they have been caregiving for longer but can feel less satisfied with the role [9,10], because ageing is associated with a negative impact from caregiving [11,12].

### Coping with caregiving

A review of the coping literature presented around 400 ways of coping [13], which can be understood in terms of the various functions they have for individuals living in challenging circumstances. For example, distraction, cognitive restructuring, minimisation and acceptance can help an individual accommodate to a stressful experience, whereas cognitive and behavioural avoidance, wishful thinking and denial can help them escape from the reality of their difficulties [13] Of these 400 ways of coping, different strategies are likely to be more, or less, helpful in a given situation. For example, a reliance on behavioural avoidance seems incompatible with the need for a caregiver to help their loved one. On the other hand, problem-focused coping and social support coping have been found to be the two most useful coping strategies for caregivers [14]. Problem-focused coping is helpful because action such as seeking information and medical assistance can aid with caring for children with additional needs [14], and has a positive impact on parents when such efforts benefit their children [15]. Social support from family, friends and healthcare professionals is associated with fewer stress-related problems and depression [16,17], and lesser feelings of social isolation [18]. However, although medical assistance, information, and social support are helpful, some families experience unmet resource and support needs, such as access to treatment and therapy [19], and information about teaching, services, and behaviour management [20]. Furthermore, social support may not be accessible because of caregiving demands [7,21]. When external resources are not available, caregivers are reliant on internal resources to help them accommodate challenges [13]. It is therefore important to evaluate the utility of coping strategies that might boost caregivers’ internal resources and help them cope with the ongoing demands of caregiving.

### Positive reappraisal coping

Positive reappraisal coping is a meaning-based cognitive coping strategy involving appreciation of the positive aspects of difficult situations (e.g., closer relationships), reinterpreting neutral information in a positive way, or comparing oneself positively with others [22], thereby “control[ing] the meaning of the problem” [23] (p.124). Cognitive efforts to derive benefit in this way are incompatible with ruminating about distressing events, and positive emotions associated with a focus on benefits are likely to improve wellbeing. Given that stressful situations cause heightened autonomic nervous system reactivity and unpleasant physical reactions, such as a racing heart and fatigue [24], positive emotions can reduce the physiological impact of feeling sad or angry [25] and provide the person with respite from negative emotions and their impact [26]. Folkman and colleagues assessed the psychological well-being of the partner caregivers of men with Acquired Immune Deficiency Syndrome (AIDS) before and after bereavement, and found that meaning-based cognitive coping efforts such as positive reappraisal were associated with positive psychological states and that those positive psychological states helped caregivers to sustain efforts to cope with the ongoing challenges [27–29]. An intervention to support this form of coping might therefore help those caring for people with ASD to experience positive psychological outcomes and help them carry on with their caregiving responsibilities.

### The Positive Reappraisal Coping Intervention (PRCI)

The PRCI is a self-help intervention, originally developed for women undergoing fertility treatment [30,31]. The PRCI comprises of ten statements promoting positive reappraisal coping, adapted from positive reappraisal coping inventories. The statements are generic, and do not refer to infertility, thus making them potentially applicable in other contexts. Infertile women found the PRCI to be acceptable [30,31] with significant benefits on anxiety [32]. The PRCI has not yet been evaluated in a caregiving context, but benefits were expected because 1) The PRCI was based on theoretical and empirical work demonstrating the benefits of this form of coping in a caregiving sample, 2) Caregiving, like infertility, involves challenges that continue over time and require ongoing accommodation, 3) Some caregivers of those with ASD naturally employ positive reappraisal coping, reporting positive aspects of caregiving such as a greater bond with their child [33], and 4) Meaning-based strategies such as benefit-finding have been associated with improved psychological well-being and lower stress in parents caring for children with ASD [34,35]. We expected that some caregivers in the present study would be familiar with positive reappraisal coping and that a greater use of this strategy would be associated with better psychological wellbeing.

### The present study

The Medical Research Council (MRC) framework [36] advocates that intervention developers assimilate a “continuum of increasing evidence” for their intervention from theory through to implementation (p. 695). Theory and evidence support positive reappraisal coping as a mechanism of intervention in the caregiving context, satisfying the first (preclinical) stage of this framework. Nevertheless, it is important to determine the effectiveness of any coping strategy by establishing its impact in a specific context [27]. Caregiving contexts vary in numerous ways such as the typical ages of the carer and care recipient, their relationship (e.g., romantic partner, child, sibling), the origin of the condition causing a need for care (e.g., congenital, developing over time), prognosis (e.g., recovery, deterioration), and the extent to which the care recipient might have any responsibility for their circumstances (e.g., genes, accident, behaviour). Such differences mean that a form of coping used and associated with positive psychological wellbeing in the specific context of caring for a romantic partner with AIDS might have different outcomes in the context of caring for a child with ASD. The Phase 1 (modelling) stage of the MRC framework recommends that intervention developers consult the intended recipients of interventions (e.g., using surveys) to maximise the likelihood that their intervention will be appropriate for the context for which it is intended [36]. In line with the recommendations of the MRC framework, the aim of the present study was to establish whether positive reappraisal coping is used by caregivers and associated with psychological wellbeing, to assess whether a PRCI is appropriate for the caregivers of those with ASD. In the present study, caregivers were asked to reflect on the 10 positive reappraisal coping items from the existing PRCI [31,32] and to write about any examples of their caregiving experience that came to mind when reading the items. They also completed measures of burden, resilience, and emotions to establish whether positive reappraisal coping was associated with better psychological wellbeing. It was expected that caregivers would write about examples of using positive reappraisal coping and that the use of such efforts would be associated with greater resilience, more positive and less negative emotions, and a lesser sense of caregiving burden.

## Method

### Ethics statement

This study was approved by the University of South Wales Faculty of Life Sciences and Education Ethics Panel [FESG1705006] prior to the study commencing. Consent was collected via an online consent form where participants endorsed ethics statements that confirmed their willingness to participate before proceeding to the survey.

### Participants

One hundred and twelve participants who considered themselves the caregivers of a person with ASD contributed to the evaluation of the PRCI items. The mean age of caregivers was 38.15 years (SD = 7.65) and the majority were white (95.50%, n = 107). Nearly three quarters of caregivers were married/cohabiting (70.50%, n = 79), and nearly all were women (97.30%, n = 98), who were maternal caregivers (93.80%, n = 105). Around one third (33.03%, n = 37) of caregivers were in paid employment and a similar proportion had graduate/postgraduate qualifications (34.82%, n = 35). Eighty-one caregivers (72.3%) were caring for one individual with ASD, with 26 (23.2%) caring for two individuals, and five (4.5%) caring for three or four. The ages of care recipients ranged from two to 46 years (mean = 11.71, SD = 7.22). Caregivers had been caring for a person with ASD for between 6 months and 31 years (mean = 9.76, SD = 5.90). Nearly two-thirds of caregivers (64.3%, n = 72) spent more than 71 hours per week on caregiving duties.

This sample represented 64.74% of those who consented to the study. Sixty-one caregivers were excluded from analyses because they did not complete all study materials. Those who were excluded were of similar age (37.83 years, SD = 7.69), ethnicity (96.50%, n = 167 were white), relationship status (68.79%, n = 119 were married or cohabiting), gender (97.1%, n = 168 were women), relationship to the care recipient (94.8%, n = 164 were maternal caregivers), employment status (32.95%, n = 57 were in paid employment) and educational background (28.90%, n = 49 had graduate or postgraduate qualifications). Of the 39 caregivers caring for more than one person, 31 (79.49%) completed all study materials, compared to 81 (60.45%) of those caring for only one person, χ^2^ (df = 1) = 4.80, p = .03. Those completing all materials were caring for slightly older people than those who did not complete all materials (mean = 11.71 years, SD = 7.21 vs. mean 9.41 years, SD = 4.13, respectively), t(171) = 2.29, p = .012, and to have been caregiving for more years (9.76, SD = 5.90 vs. 8.34, SD = 5.03, although this difference only approached significance, t(171) = 1.61, p = .06).

### Materials

All measures were presented using Survey Monkey online survey software (www.surveymonkey.com).

#### The PRCI evaluation measure

This measure was developed by the first author to elicit free-text responses to PRCI items and established whether caregivers reported the use of positive reappraisal coping. Caregivers were provided with written guidance which asked them to think about their experiences of caring for somebody with ASD in terms of what aspects of caregiving were perceived as positive or to have benefit (e.g., to the caregiver and/or the person receiving care) and what thoughts helped them to continue with caregiving, even when it was difficult. Reassurance that there was no assumption of positive experiences was provided.

The 10 positive reappraisal coping items from the existing PRCI [31,32] were preceded by the lead statement: When caregiving I….

1. Focus on the benefits and not just the difficulties.
2. Try to think more about the positive things in my life.
3. Try to do something meaningful.
4. Learn from the experience.
5. See things positively.
6. Make the best of the situation.
7. Look on the bright side of things.
8. Find something good in what is happening.
9. Try to do something that makes me feel positive.
10. Focus on the positive aspects of the situation.

Unlimited space was provided next to each PRCI item for caregivers to write whatever came to mind when they considered each item. Responses were coded as: 1) an example of a focus on the positive that was indicative of positive reappraisal coping, 2) a negative response, or 3) a response that was neutral or ambiguous with regards to coping or emotional valence. Responses to PRCI items were coded by the second author (JH) and verified by the first and fourth authors (DL and AC). Instances of coding disagreement were resolved on discussion until 100% agreement was achieved.

#### The Brief Resilience Scale (BRS) [37]

This six-item self-report measure comprises of three positively and three negatively framed items assessing the ability to “bounce back or recover from stress” [38, p.194]. Responses are made on a 5-point scale ranging from 1 (strongly disagree) to 5 (strongly agree). Negatively framed items are reversed scored, before scores are summed and divided by six. Cut off scores for resilience are: 1–2.99 (low resilience), 3–4.30 (normal resilience) and 4.31–5 (high resilience; 39). Cronbach’s alpha for the BRS ranged between .80-.91 in groups of students, women with or without fibromyalgia, and cardiac rehabilitation patients, and mean scores ranged between 3.53 and 3.98 [38]. Convergent validity was demonstrated by positive relationships between resilience and factors associated with positive psychological wellbeing (e.g., positive affect, positive reframing) and negative relationships with factors linked to psychological difficulty (e.g., anxiety, depression, negative social interactions) [39]. In the present study, α = .90.

#### The Caregiving Burden Inventory (CBI) [38]

The CBI has 24-items assessing caregiving demands, yielding a Total Burden scale and five subscales assessing different burdens: Time Dependence (e.g., demands on caregiver’s time), Developmental (e.g., not feeling at the same developmental stage as peers), Social (e.g., interpersonal issues), and Emotional (e.g., negative feelings about care recipient). Each subscale has 5 items except the Physical burden subscale (e.g., fatigue, consequences of caregiving for physical health), which has 4 items. Participants rated items on a four-point scale anchored 0 (never) to 4 (nearly always). Subscale scores are summed with the 4-item Physical Burden scale weighted by a factor of 1.25, and Total Burden is calculated by summing subscale totals. Higher scores represent greater burden. Novak and Guest report Cronbach’s Alphas between .73 and .86 for the 5 subscales [40]. Means for subscales ranged from 5.1 (Emotional, Social) to 10.1 (Time Dependence) in parents caring for children with Paediatric Acute-onset Neuropsychiatric Syndrome, with a mean for Total Burden of 36.7. In that study, mothers who had to reduce employment hours, had a child who had to change schools or who had missed days of school reported significantly higher Total Burden than those who did not have such challenges [39], supporting the external validity of the CBI in parents caring for children with additional needs. In the present study, Cronbach’s Alphas were good: α = .84 Time Dependence) α = .78, (Emotional), α = .80, (Physical), α = .85 (Developmental), α = .77 (Social) and α = .87 for the Total Burden scale.

#### Emotional wellbeing: The emotional reactions subscale from the Daily Record Keeping sheet (DRK) [40]

Participants rated each of eight words assessing common positive emotions (happy, fulfilled, content, encouraged, positive, hopeful, relieved, confident; maximum score = 24) and 12 words assessing common negative emotions (tense, angry, nervous, sad, disappointed, discouraged, anxious, unsure, hesitant, doubtful, uncertain, worried; maximum score = 36), according to the extent to which they had experienced that emotional reaction on the previous day of caregiving. Responses were made on a 4-point scale anchored 0 (not at all) to 3 (a great deal), and scores for positive and negative emotions were summed separately. The DRK assesses common emotional reactions and has been extensively used to track emotions during fertility treatment. It is sensitive to the changes in positive and negative emotions that would be expected over the course of fertility treatment [41]. Reliability coefficients for DRK subscales range from 0.76 - .82, depending on subscale [42]. In the present study, emotional wellbeing subscales demonstrated good internal consistency α =. 88 and α = .91 (Positive and Negative Emotions, respectively).

### Procedure

Advertisements inviting participation from caregivers of a person with ASD were placed on relevant Facebook groups. Those who accessed the survey were provided with an online information sheet and consent form and after confirming consent were able to access and complete the online study materials. Following submission of materials, participants received thanks for participating and an online debrief. Caregivers received no remuneration.

### Data analysis

The required sample size of 84 participants was determined by G*power analysis [43] for a medium effect size (Cohen’s f^2^ = 0.3), statistical power .80 and probability of < .05, two tailed. Quantitative analyses were conducted using the statistical software packages SPSS [44] and RStudio [45] and p < .05 was classed as significant. Screening analyses computed using the frequencies and descriptive commands showed psychological variables were normally distributed [46]. Skewness coefficients (Standard Error = .23) were: -.02 (BRS), .18 and -.25 (Positive and Negative Emotions, respectively), and -.61 (Time Dependence), .43 (Emotional) -.42, (Physical), -.40 (Developmental), -.11 (Social), and -.39 (Total Burden). Kurtosis (Standard Error = .45) was: -.26 (BRS), -.74 and -.37 (Positive and Negative Emotions, respectively), and -.27 (Time Dependence), -.60 (Emotional), .10 (Physical), -.29 (Developmental), -.48 (Social), and .93 (Total Burden). Descriptive statistics were computed for age, characteristics of the caregiving context, resilience, positive and negative emotions, and caregiving burden.

Content analysis was conducted, following the analytical steps of Krippendorff [47]:

1. Unitising the data: Sampling units were identified from which representation of the data was made (participants’ written utterances).
2. Sample selecting: The data set was of moderate size and all texts were included.
3. Coding: Responses were coded as 1) examples of positive reappraisal coping 2) negative responses, 3) neutral responses.

Coded data were summed to give category totals. Chi-square goodness of fit analyses were used to assess differences in the frequency of responses categorised as Positive Reappraisal Coping, Negative, or Neutral for each PRCI item. Significant omnibus Chi-square analyses were followed up with pairwise comparisons of positive reappraisal coping, negative, and neutral responses, with a Bonferroni correction to control the familywise error rate, using RStudio software [45]. Pearson’s correlational analyses were used to examine zero-order relationships between positive reappraisal coping, negative or neutral responses to PRCI items, and other continuous study variables. The number of hours spent caring each week was collected in 10-hour categories from zero to 71+ hours per week but examination of the distribution showed that 72 participants cared for 71+ hours with few participants endorsing the other time categories. This variable was dichotomised into ≤ 70 hours and 71+ hours, and independent samples t-tests were used to establish whether the number of examples of positive reappraisal coping, negative responses, or neutral responses to PRCI items differed between groups.

Thematic Analysis [48] using an inductive approach was undertaken to analyse the qualitative data from the PRCI statements (in the Positive Reappraisal, Negative, and Neutral categories), using the following steps; Reading and familiarisation with scripts, theme searching, reviewing identified themes and defining/naming themes. Researcher AC identified initial themes while researcher DL independently verified themes and subthemes. Agreement regarding the identified themes was reached on discussion between these researchers

## Results

**Fig 1:**
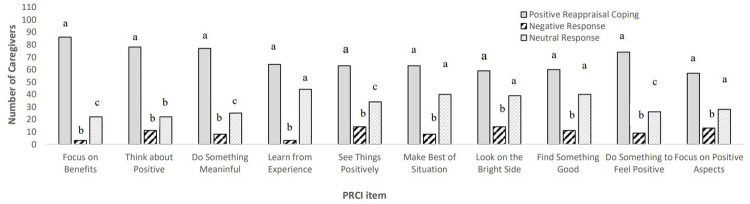
Number of participants providing examples of positive reappraisal coping, negative responses, or neutral responses to PRCI items.

On average, participants provided examples of positive reappraisal coping to 6.10 (SD = 3.68) of the 10 PRCI items, negative responses to .84 items (SD = 1.69) neutral responses to 2.91 items (SD = 3.14) and did not respond to .14 items (SD = .60). Positive reappraisal coping responses comprised of 13.25 words on average (range 1–448), negative responses averaged 16.43 words (range 1–337) and neutral responses contained an average of 3.80 words (range 1–40). There were significant differences in the frequency of examples of positive reappraisal coping, negative responses, and neutral responses (see Table 1). There were significantly more frequent examples of positive reappraisal coping than negative responses for every item, and significantly more frequent examples of positive reappraisal coping than neutral responses for four items (see Fig 1). The item generating examples of positive reappraisal coping from the greatest number of participants was the item “Focus on the benefits and not just the difficulties”, for which 86 caregivers (76.79%) generated examples, and the item generating the fewest was “Focus on the positive aspects of the situation” with examples provided by 57 caregivers (50.89%). The item generating the greatest number of negative responses caregivers (n = 14, 12.50%) were “See things positively” and “Look on the bright side of things” and the items generating the fewest were “Focus on the benefits and not just the difficulties” and “Learn from the experience” (n = 3, 2.68%). The greatest number of neutral responses were seen to the item “Learn from the experience” (n = 44, 39.29%) and the fewest for the items “Focus on the benefits and not just the difficulties”, and “Try to think more about the positive things in my life” (n = 22, 19.64%). Nil responses were few (n = 1 for 5 items and n = 4 for 1 item).

**Table 1:** Chi-square goodness of fits statistics comparing positive reappraisal coping, negative responses and neutral responses to PRCI items.

PRCI item	χ2 (df = 2)
Focus on the benefits and not just the difficulties	100.52***
Try to think more about the positive things in my life	68.38***
Try to do something meaningful	68.05***
Learn from the experience	52.20***
See things positively	32.38***
Make the best of the situation	41.05***
Look on the bright side of things	27.23***
Find something good in what is happening	32.70***
Try to do something that makes me feel positive	59.38***
Focus on the positive aspects of the situation	26.80***

### Psychological wellbeing

As shown in Table 2, caregivers’ resilience was at the upper threshold of the ‘low resilience’ category [49], and mean scores for negative emotions were in the mid-range. Their total burden scores were nearly 20 points higher than those reported by parents caring for children with PANS [39]. Greater resilience, higher positive and lower negative emotions, and less burden was reported by caregivers who responded to more PRCI items with examples of positive reappraisal coping. Caregiver age and years of caregiving were not associated with positive reappraisal coping. Caregivers providing negative responses to more PRCI items had been caring for more years and a trend towards more frequent negative responses to PRCI items was seen in older caregivers. Those who generated negative responses to more PRCI items were less resilient, and reported more negative emotional states, greater total burden, and higher emotional, physical, and developmental burdens. A greater number of neutral responses to PRCI items were associated with more negative emotions, greater physical burden, and a trend towards a higher developmental burden, but was unrelated to their age and years of caregiving. There were no significant differences in positive reappraisal coping, *t*(110) = .86, *p* = .39, negative responses, *t*(110) = -1.28, *p* = .21, or neutral responses, *t*(110) = -.28, *p* = .78, between those caring for <70 or 71+ hours per week.

**Table 2:** Zero-order correlations between responses to PRCI items and caregiver characteristics, resilience, emotional wellbeing, and caregiver burden.

	Mean (SD)	Positive Reappraisal coping	Negative responses	Neutral Responses
**Caregiver age**	38.15 (7.65)	-.03	.17^t^	.06
**Number of years of caregiving**	9.76 (5.70)	-.09	.22*	.01
**Resilience (5)**	2.99 (0.89)	.23*	-.23*	-.15
**Positive Emotions (24)**	9.59 (4.84)	.20*	-.42***	.01
**Negative Emotions (36)**	17.81 (7.94)	-.29**	.20*	.22*
**Time Dependence (20)**	15.18 (3.54)	-.17^t^	.13	.09
**Emotional (20)**	5.34 (3.57)	-.25**	.26**	.14
**Physical (20)**	13.05 (4.26)	-.28**	.20*	.21*
**Developmental (20)**	12.19 (4.42)	-.35***	.37***	.18^t^
**Social (20)**	9.83 (4.60)	-.17^t^	.14	.10
**Total (100)**	55.58 (13.78)	-.36***	.33***	.02

Note. t p < .10

* p < .05

** p < .01

*** p < .001.

### Qualitative analysis of PRCI statements

Following Thematic Analysis, several themes and subthemes were identified (see Table 3). Results are presented using verbatim extracts.

**Table 3:** Identified categories, themes and subthemes from Thematic Analysis.

Category	Theme	Subtheme
Positive	Use of PR coping	Reflection on the positives
		Hard but rewarding
		Gratitude
	Child’s achievements	Child’s progress
		Being the reason they succeed
		Appreciation of the child
		Failure leads to success
	Caregiver adaptation	Continual learning
		New ways of coping
		Resilience
Negative	Pessimism	There are few positives
		Lack of choice and control
	Mental and physical exhaustion	
	Unmet needs	Professional support
		Fight for diagnosis and support
		Social support

Note: PR = positive reappraisal; “positive” indicates category where participants provided positively framed responses to PRCI statements; “negative” indicates category where participants provided negative responses to PRCI statements.

#### Positively framed responses theme 1: The use of positive reappraisal coping

Caregivers reported using Positive Reappraisal (PR) coping techniques when caregiving. Subthemes were: ‘Reflection on positives’, ‘hard but rewarding’ and ‘gratitude’. Responses suggested that caregivers were cognitively reframing the caregiving situation to view it in a more positive light [50]:

*Reflection on positives*. Responses indicate that caregivers are aware of the demands of caregiving but are actively identifying and focusing on positive aspects of the caregiving experience, in line with the principles of positive reappraisal coping.

“*Caring can involve lots of setbacks*, *difficult experiences–you have to try and dismiss those and focus on the good”–*REF 050

*Hard but rewarding*. Caregivers’ responses also suggested that the difficulties of caregiving are offset by the rewards they perceive:

***“****It is extremely hard but also very rewarding*. *It is so important to take time out for yourself if you possibly can to recharge your batteries*.*”–*REF 082

Participants spoke of a generic reward from caregiving and of small triumphs:

***“****It’s the most rewarding thing I will ever do she’s my daughter and it’s the little things and obstacles we overcome that make it so rewarding”–*REF 357

*Gratitude*. Caregivers wrote about their appreciation of positive aspects of family life and the support they receive:

*“Definitely*. *I am so lucky*. . .*to have an amazing husband*, *beautiful kids*, *our own home*. *I am very lucky”–*REF 044

*“Extra help from teachers at school to help her excel such as homework”–*REF 083

These responses indicate active, meaning-based cognitive efforts [51] that involve them identifying and deriving benefit from positive aspects of life in general and caregiving in particular.

#### Positively framed responses theme 2: Child’s achievements

This theme encompassed caregivers’ views that the achievements of their child provided an opportunity to reflect positively on their role as caregivers. This theme has four subthemes: ‘Child’s progress’, ‘Being the reason they succeed’, “Appreciation of the child” and ‘Failure leads to success’.

*Child’s progress*. Responses referred to the development or progress of the child:

*“The little bit of progress we make cheers me up to some extent”–*REF 029

*“I focus on the achievements of my son and all the hard work we put in to achieve that”–*REF 043

These responses indicate that caregivers appreciated the value of their child’s progress, even if this progress is small and hard won.

*Being the reason their loved ones succeed*. Other responses more emphatically demonstrated caregivers’ recognition that they were a key source of impetus facilitating the past, present, and future achievements of their child:

*“My boys have achieved so much because of me*.*”-* REF 002.

*“Am proud I have made changes to his education to ensure he has the best available*.*”*–REF 198

In line with the findings of Ryan and Cole [52] who found that mothers of children with ASD facilitate their social interaction and skills, these responses communicate caregivers’ focus on their pride in knowing their own efforts benefit their children’s development.

*Appreciation of the child*. A number of responses also communicated a clear appreciation of their child’s personality:

“*That my son is very creative and loving”–*REF 358

Such responses reflect the caregiver’s focus on the positive aspects of their child as an individual, independent of their diagnosis and difficulties. Despite the stress of caregiving, these caregivers were actively focusing on their child ‘as a person’, which brought them joy.

*Failure leads to success*. Some caregivers said they encouraged their children (and themselves) to view unsuccessful efforts as ‘not a failure’:

*“We celebrate the achievements*, *no matter how small*, *and we find better ways of dealing with things that didn’t go right first time*. *There’s always a positive somewhere*, *even if sometimes it’s hard to find it straight away*.*”–*REF 521

Such responses suggest efforts to focus on the potential for success in difficult and unrewarding circumstances rather than on the negative consequences of setbacks.

#### Positively framed responses theme 3: Caregiver adaptation

This theme reflects the positive nature of caregiver learning, adaptation, and development. Caregivers showed awareness of a need to adapt and that caregiving is a continual learning process. This theme has the following subthemes: ‘Continual learning’, ‘New ways of coping’, and ‘Resilience’.

*Continual learning*. In response to the statement ‘Learn from the experience’, caregivers said they were learning and adapting continuously, using the information gained through trial and error to cope with the ongoing demands of caregiving:

*“I have made so many mistakes along the way and learnt from every single one*.*”–*REF 046

These responses convey a positively toned stance on caregivers’ own learning, rather than seeing mistakes and the need to learn as an indication of failing on their part. Caregivers also reported continually developing new coping strategies, based on information gained from their experience as a caregiver.

*“I learn new things all the time and new coping strategies “*.–REF 037

These findings indicate that caregivers accept that their role requires a flexible approach to coping with caregiving and the development of new ways of managing caregiving demands. This aligns with Lazarus and Folkman’s proposals that coping requires constantly changing behavioural and cognitive efforts to meet ongoing demands [51].

*Resilience*. This subtheme reflects caregivers’ awareness of their determination and ability to ‘bounce back’, and seek the positives, even in the face of demands and difficult experiences:

*“…I throw myself into being the best parent I can be (often I fail) and take on each day with a positive attitude no matter how disastrous the previous day was*. *I can concentrate on my own life and career when the children are older…”* REF 221

Such responses suggest that caregivers endeavour to carry on and keep going as best they can, demonstrating resilience and determination.

#### Negatively framed responses theme 1: Pessimism

The theme of pessimism incorporated three subthemes: ‘There are few positives’, ‘Lack of choice and control’, and ‘mental and physical exhaustion’.

*There are few positives*. Some caregivers responded to PRCI statements by indicating that there were few to think about:

*“There is not much of a bright sight*. *not for me and my family”—*REF 007

*“Don’t have much positives to focus on my kids see me through”*–REF 124

Other caregivers emphasised that there were no positive aspects of having a child with ASD. In response to “I will: find something good in what is happening”, one participant commented:

*“I don’t*. *What good comes out for my disabled child*? *For me as a parent carer and for my other child who is immensely stressed out by her sibling*.*”*–REF 008.

Although these caregivers identify few, if any, positive aspects of having a child with ASD, the phrase “my kids see me through” still presents a positive focus regarding the role her children have in helping her to tolerate difficult circumstances. Some caregivers seemed to focus more on the ASD diagnosis than on the caregiving experience per se. Such a specific focus might reduce their awareness of the wider caregiving context and any positive aspects that could give them respite from challenges that upset them.

*Lack of choice and control*. Caregivers expressed negative thoughts about the lack of choice in the caregiving context. When responding to the statement of ‘Try to do something meaningful’, one participant stated:

“I care because I have to. I don’t have a choice (child with ASD and severe learning diffs). I cannot relate to the question.”–REF 003,

This sense of a lack of choice perhaps interferes with the identification of positive aspects of the caregiving role, findings which are in line with previous research where caregivers (often parents) reported a lack of control and a sense of helplessness [4,13].

#### Negatively framed responses theme 2: Mental and physical exhaustion

This theme illustrates feelings of being worn out and exhausted:

*“It’s hard & is a full-time job with no breaks it’s mentally & physically exhausting”*.–REF 025.

When responding to the statement of ‘focusing on the positive’ one participant poignantly highlighted the relentless nature of the caregiving role for some caregivers:

*“I really don’t*. *As I said*, *I am tired and worn out and I know it will only stop the day I die*.*”*–REF 010

Such mental and physical fatigue from caregiving has been reported by other mothers of children with ASD [53].

#### Negatively framed responses theme 3: Unmet needs

This theme of unmet related to professional, social, and educational needs and included the subthemes: ‘Professional support’, and ‘Social support’.

*Professional support*. Caregivers’ spoke of an ongoing struggle to obtain professional support for their child.

*“It is very difficult and you have to fight for support in an underfunded system as well as carrying out your caring role*. *No wonder we are all exhausted*. *Living with someone with ASD affects the whole family*.*”-* REF 132.

These responses indicate ongoing battles to obtain appropriate care and support, in line with the findings of Chiri and Warfield [19]. Such struggles seem to outweigh thoughts of the child with ASD causing problems:

*“It is not my son that causes me the most ’stress’ it is the ignorance of society*, *fighting for diagnosis*, *fighting for educational interventions—it’s exhausting*.*”–*REF 016.

*Social support*. Alongside a lack of professional support, loneliness and social isolation were expressed by caregivers:

*“It can be very isolating socially and sometimes it’s a circle that can’t be broken*.*”–*REF 062 *“that it is down to me only for my son*, *no family comes to help”*.–REF 105

The findings demonstrate a sense that the caregivers feel abandoned by professionals [19,20], family and friends, as has been reported previously [7,17,19,22].

## Discussion

The challenges of caregiving for those with ASD have been extensively documented [1,2,54], and were evident in the present study where caregivers reported low resilience and a high time dependency burden, as well as a greater overall burden than parents caring for children experiencing psychiatric crisis [39]. The time dependency burden is unsurprising given that nearly two-thirds of participants in the present study spent more than 71 hours on caregiving duties each week. In line with the recommendations of the MRC framework, the aim of the present study was to establish whether positive reappraisal coping strategies were used and associated with greater psychological wellbeing, prior to the development of a future self-help PRCI for the caregivers of those with ASD. The results showed that when caregivers were invited to write about what “comes to mind” from their caregiving experience in response to PRCI items, examples of positive reappraisal coping were more frequently generated than negative responses. Furthermore, positive reappraisal coping was related to more resilience, more positive and less negative emotions, and a lesser sense of caregiving burden. The results suggest that people caring for those with ASD use positive reappraisal strategies, and that these are associated with positive psychological outcomes, in line with the findings of Folkman and colleagues [27–29]. There was some variability in the emotional valence of caregivers’ responses to PRCI items, however, and not all caregivers reported positive reappraisal coping. Negative responses were associated with lower resilience and less positive emotions on one hand, and with more negative emotions and a greater sense of caregiver burden on the other. Given the evident link between positive reappraisal coping and psychological wellbeing in the present study, development of a PRCI to support caregivers in the effective use of such coping efforts could be a valuable means of supporting the psychological wellbeing of the caregivers of those with ASD. The MRC framework emphasises that intervention developers should establish key aspects of interventions such as the mechanisms of intervention, acceptability, and barriers to implementation. Further development and evaluation of the PRCI will be in line with these guidelines.

### Mechanisms and moderators

Lazarus and Folkman [55] define cognitive appraisals as “cognitive manoeuvres” that change the meaning of a situation. The cognitive manoeuvres involved in positive reappraisal coping can be understood as a form of ‘mental gymnastics’ involving efforts to appreciate any positive elements of a challenging situation. Such efforts underpin other phenomena associated with positive outcomes in difficult circumstances, such as posttraumatic growth [53], and are included in some Positive Psychology Interventions (PPIs) associated with mental health benefits [54]. The association between positive reappraisal coping and positive emotion in the present study suggests that this form of coping might increase positive emotions via the cognitive processes involved in deriving benefit from challenging experiences [26,49]. However, study variables were assessed concurrently and there are reciprocal relationships between coping, appraisal, and emotion [55,56], meaning that we cannot ascertain causality between positive reappraisal coping and psychological wellbeing in the present study.

In addition, attrition in the present study meant it was not possible to establish whether caregivers who did not complete the study used positive reappraisal coping and whether it was related to psychological wellbeing if they did. Those caring for two or more people with ASD, who had been caregivers for more years, and whose care recipient(s) were older were more likely to have responded to the PRCI evaluation. We also noted that some examples of positive reappraisal coping were characterised by greater service provision and support and were less likely to emphasise unmet needs or depressed states of mind than negative comments. Non-engagement with the PRCI evaluation and/or positive reappraisal coping may be explained by circumstances that affected the ability or willingness of participants to do so. Moreover, more than 96% of participants were of white ethnicity, meaning that the perspectives of caregivers from other ethnic backgrounds should be explored further. Finally, some responses to PRCI items suggested that positive reappraisal in the caregiving context is more challenging for some than for others. Future studies should therefore aim to determine factors that could influence the use and utility of the PRCI amongst caregivers.

One consideration for future development and evaluation of the PRCI is whether examples of positive reappraisal coping in action reflect appreciation of circumstances which are unsatisfactory. The extent to which a positive focus in difficult times is illusory or exaggerated was discussed in detail by Coyne and Tennen [57], who cite reports of benefits in health challenges, trauma, and disaster, and debate whether these reflect genuinely positive outcomes. We could not determine whether examples of positive reappraisal coping reflect experiences that are actually better than those reporting negative responses because we did not enquire about the difficulties with relationships, finances, and mental health reported by caregivers in past research [1,2,54], nor about the nature of the ASD diagnosis and the prognosis for the care recipient [6–8]. Such factors impact on the wellbeing of caregivers and potentially on their use of positive reappraisal coping. Given that mental and physical exhaustion and unmet needs were spontaneously reported by caregivers, it is important we ensure that the positive focus encouraged by the PRCI does not mean that caregivers ignore problems or disengage from efforts to resolve deficits in their circumstances. Future evaluations of the PRCI should include prospective, randomised controlled studies and include factors such as diagnosis, prognosis, and personal circumstances [58] to fully understand the extent to which the PRCI might be beneficial.

### Development of a PRCI for the caregivers of those with ASD

Although the present study suggests that caregivers of those with ASD use positive reappraisal coping strategies, and that these are linked with positive psychological wellbeing, variability in responses suggest that some caregivers do not recognise positive aspects in their caregiving role or feel these are so minor that they are outweighed by negative factors. Such an interpretation of their circumstances might be entirely veridical, but an alternative explanation is that there are meaningful and positive aspects of their caregiving experiences, which are not recognised or appreciated. Furthermore, some caregivers might have misunderstood the principle of positive reappraisal coping in the caregiving context, interpreting the strategy as relating to what is good about loved ones having ASD (e.g., “I can’t really see the benefits of having autistic kids”–REF 018) rather than what might be the positive aspects of their caregiving role. We presented caregivers with the positive reappraisal coping items from the PRCI but did not provide information about what positive reappraisal ‘is’, nor the potential benefits. Caregivers could benefit from information about cognitively reframing their caregiving experiences and from examples from caregivers in the present study. In evaluations of the PRCI during fertility treatment, women received a leaflet presenting PRCI items, information about the challenges of treatment and positive reappraisal coping, and instructions for the use of the intervention [32]. Development of the PRCI for caregivers will involve the inclusion of a leaflet tailored to this context, and focus groups will be used explore the use and effects of the intervention, in line with a MRC framework informed evaluation of a self-help distraction coping intervention [59]. This exploration will facilitate further modelling of the PRCI. In the present study, no significant differences were found between neutral responses and examples of positive reappraisal coping for several items. Neutral responses included too little information to categorise the response as demonstrating positive reappraisal or a negative response (e.g., I’m a different person to the one I was before I became a mother” [REF 001]). One limitation of our online methodology is that it did not permit us to explore neutral responses to gauge the valence of reactions to PRCI items, and focus groups would facilitate this.

## Conclusions

This MRC framework informed modelling study suggests that positive reappraisal coping is used by the caregivers of those with ASD and is associated more positive emotion and a lesser sense of caregiving burden. This link with positive emotion suggests the PRCI is a form of PPI that might promote positive emotions and thereby support the individual’s wellbeing [58], although further evaluation is needed to confirm this. The advantage of the planned brief, self-help PRCI is that it will be economical to produce and afford considerable flexibility to the caregiver because it can be used whenever there is the opportunity and need. This practical advantage may be particularly important to caregivers who are not able to attend face-to-face therapy or support provision. Moreover, unlike other coping strategies found helpful in the caregiving context, such as problem-focused and support-seeking strategies [60] the caregiver is not dependent on the contribution of others to utilise the strategy. Rigorous evaluation of the PRCI in line with the MRC framework [37] is essential, however, to help us to understand the mechanisms and moderators of PRCI effects [58] and to answer criticisms about the quality of scientific evidence regarding the nature of positive psychological factors in difficult circumstances [57].

## References

[1] Depape AM, Lindsay S. Parents’ experiences of caring for a child with autism spectrum disorder. Qual Health Res. 2015 Apr 16;25(4):569–83. doi: 10.1177/1049732314552455 25246329

[2] Stoner JB, Stoner CR. Career Disruption: The Impact of Transitioning from a Full-Time Career Professional to the Primary Caregiver of a Child with Autism Spectrum Disorder. Focus Autism Other Dev Disabl. 2016;31(2):104–14.

[3] Hartley S. L., Barker E. T., Seltzer M. M., Floyd F., Greenberg J., Orsmond G., et al. The relative risk and timing of divorce in families of children with an autism spectrum disorder. J Fam Psychol. 2010;24(4):449. doi: 10.1037/a0019847 20731491 PMC2928572

[4] Baker D. L., & Drapela LA. Mostly the mother: Concentration of adverse employment effects on mothers of children with autism. Soc Sci J. 2010;47(3):578–92.

[5] Ekas N V., Timmons L, Pruitt M, Ghilain C, Alessandri M. The Power of Positivity: Predictors of Relationship Satisfaction for Parents of Children with Autism Spectrum Disorder. J Autism Dev Disord. 2015 Jul 19;45(7):1997–2007. doi: 10.1007/s10803-015-2362-4 25601217

[6] Lyons A. M., Leon S. C., Phelps C. E. R., & Dunleavy AM. The impact of child symptom severity on stress among parents of children with ASD: The moderating role of coping styles. J Child Fam Stud. 2010;19(4):516–24.

[7] Bromley J., Hare D. J., Davison K., & Emerson E. Mothers supporting children with autistic spectrum disorders: Social support, mental health status and satisfaction with services. Autism. 2004;8(4):409–23. doi: 10.1177/1362361304047224 15556959

[8] Hastings RP. Child behaviour problems and partner mental health as correlates of stress in mothers and fathers of children with autism. J Intellect Disabil Res. 2003;47(5):231–7. doi: 10.1046/j.1365-2788.2003.00485.x 12787155

[9] Burke M, Heller T. Individual, parent and social-environmental correlates of caregiving experiences among parents of adults with autism spectrum disorder. J Intellect Disabil Res. 2016 May 1;60(5):401–11. doi: 10.1111/jir.12271 27120984

[10] Caldwell J., & Heller T. Management of respite and personal assistance services in a consumer‐directed family support programme. J Intellect Disabil Res. 2003;47(5):352–67. 12787166 10.1046/j.1365-2788.2003.00496.x

[11] Navaie-Waliser M., Feldman P. H., Gould D. A., Levine C., Kuerbis A. N., & Donelan K. When the caregiver needs care: the plight of vulnerable caregivers. Am J Public Health. 2002;92(3):409–13. doi: 10.2105/ajph.92.3.409 11867321 PMC1447090

[12] Anna J. Esbensen MMS. Accounting for the “Down Syndrome Advantage.” Am J Intellect Dev Disabil. 2011;116(1):3–15. doi: 10.1352/1944-7558-116.1.3 21291307 PMC3071600

[13] Skinner E. A., Edge K., Altman J., & Sherwood H. Searching for the structure of coping: a review and critique of category systems for classifying ways of coping. Psychol Bull. 2003;129(2). doi: 10.1037/0033-2909.129.2.216 12696840

[14] Pain H. Coping with a child with disabilities from the parents’ perspective: the function of information. Child Care, Heal Dev. 1999;25(4):299–313. doi: 10.1046/j.1365-2214.1999.00132.x 10399034

[15] Shivers CM, Krizova K, Lee GK. Types of strain among family members of individuals with autism spectrum disorder across the lifespan. Res Dev Disabil. 2017 Sep 1;68:42–51. doi: 10.1016/j.ridd.2017.07.003 28735161

[16] Gill M. J., & Harris SL. Hardiness and social support as predictors of psychological discomfort in mothers of children with autism. J Autism Dev Disord. 1991;21(4):407–16. doi: 10.1007/BF02206867 1778957

[17] Gray D. E., & Holden WJ. Psychosocial well-being among the parents of children with autism. Australia and New Zealand Journal of Developmental Disabilities. Aust New Zeal J Dev Disabil. 1992;18(2):83–93.

[18] Dunn M. E., Burbine T., Bowers C. A., & Tantleff-Dunn S. Moderators of stress in parents of children with autism. Community Ment Health J. 2001;37(1):39–52. doi: 10.1023/a:1026592305436 11300666

[19] Chiri G., & Warfield ME(2012). Unmet need and problems accessing core health care services for children with autism spectrum disorder. Matern Child Health J. 2012;16(5):1081–91. doi: 10.1007/s10995-011-0833-6 21667201

[20] Ellis J. T., Luiselli J. K., Amirault D., Byrne S., O’Malley-Cannon B., Taras M. WJ et al. Families of children with developmental disabilities: assess-ment and comparison of self-reported needs in relation to situational variables. J Dev Phys Disabil. 2002;14:191–202.

[21] Kiami S. R., & Goodgold S. Support needs and coping strategies as predictors of stress level among mothers of children with autism spectrum disorder. Autism Res Treat. 2017. doi: 10.1155/2017/8685950 29435368 PMC5757090

[22] Thompson SC. Finding positive meaning in a stressful event and coping. Basic Appl Soc Psych. 1985;6(4):279–95.

[23] Park C. L., & Folkman S. Meaning in the context of stress and coping. Rev Gen Psychol. 1997;1(2):115–44.

[24] Pennebaker J. W., Gonder‐Frederick L., Stewart H., Elfman L., & Skelton JA. Physical Symptoms Associated with Blood Pressure. Psychophysiology,. 1982;19(2):201–10. doi: 10.1111/j.1469-8986.1982.tb02547.x 7071299

[25] Fredrickson BL, Levenson RW. Positive Emotions Speed Recovery from the Cardiovascular Sequelae of Negative Emotions. Cogn Emot. 1998. doi: 10.1080/026999398379718 21852890 PMC3156608

[26] Lazarus R. S., Kanner A. D., & Folkman S. EMOTIONS: A COGNITIVE–PHENOMENOLOGICAL ANALYSIS. In: Emotion: Theory, Research, and Experience. 1st ed. 1980. p. 189–217.

[27] Folkman S. Positive psychological states amd coping with severe stress. Soc Sci Med. 1997;45(8):1207–21. doi: 10.1016/s0277-9536(97)00040-3 9381234

[28] Folkman S, Moskowitz JT. Positive affect and the otherside of coping. Am Psychol. 2000;55(6):647. doi: 10.1037//0003-066x.55.6.647 10892207

[29] Moskowitz J. T, Folkman S. Collette L. Vittinghoff E. Coping and mood during AIDS related caregiving and bereavement. Ann Behav Med. 1996;18(1):49–57. doi: 10.1007/BF02903939 24203643

[30] Lancastle D S. Reasons to be cheerful, Part I, II, III; dispositional optimism, positive reappraisal coping and positive (re) appraisals of the situation: effects on emotional well-being and physical outcomes. 2006.

[31] Lancastle D, Boivin J S. A feasibility study of a brief coping intervention (PRCI) for the waiting period before pregnancy test during fertility treatment. Hum Reprod. 2008;23(10):2299–307. doi: 10.1093/humrep/den257 18628259

[32] ockhuijsen H. van den Hoogen A. Eijkemans M. Macklon N. Boivin J. Clarifying the benefits of the positive reappraisal coping intervention for women waiting for the outcome of IVF. Hum Brain Mapp. 2014;29(12):2712–8. doi: 10.1093/humrep/deu253 25316451

[33] Hock R. M. Timm T, M. Ramisch JL. Parenting children with autism spectrum disorders: a crucible for couple relationships. Child Fam Soc Work. 2011;17(4):406–15.

[34] Lovell B, Moss M, Wetherell M. The psychosocial, endocrine and immune consequences of caring for a child with autism or ADHD. Psychoneuroendocrinology. 2012 Apr;37(4):534–42. doi: 10.1016/j.psyneuen.2011.08.003 21889267

[35] Timmons L, Ekas N V. Giving thanks: Findings from a gratitude intervention with mothers of children with autism spectrum disorder. Res Autism Spectr Disord. 2018 May 1;49:13–24.

[36] Campbell N. C., Murray E., Darbyshire J., Emery J., Farmer A., Griffiths F., et al. Designing and evaluating complex interventions to improve health care. Br Med J. 2007;334(7591):455–9. doi: 10.1136/bmj.39108.379965.BE 17332585 PMC1808182

[37] Smith BW, Dalen J, Wiggins K, Tooley E, Christopher P, Bernard J. The brief resilience scale: Assessing the ability to bounce back. Int J Behav Med. 2008 Jul;15(3):194–200. doi: 10.1080/10705500802222972 18696313

[38] Novak M GC. Application of a multidimensional caregiver burden inventory. Gerontologist. 1989;29(6):798–803. doi: 10.1093/geront/29.6.798 2516000

[39] Farmer C, Thienemann M, Leibold C, Kamalani G, Sauls B, Frankovich J. Psychometric evaluation of the caregiver burden inventory in children and adolescents with PANS. J Pediatr Psychol. 2018;43(7):749–57. doi: 10.1093/jpepsy/jsy014 29547961 PMC6054236

[40] Boivin J, §ii MA*, Takefman JE, Brender W. Reactions to infertility based on extent of treatment failure*t*. Vol. 63, American Society for Reproductive Medicine. 1995. 7890066

[41] Boivin J, Lancastle D. Medical waiting periods: imminence, emotions and coping. Womens Health (Lond Engl). 2010;6(1):59–69. doi: 10.2217/whe.09.79 20088730

[42] Boivin J. The Daily Record Keeping Chart: Reliability and validity. In: British Psychological Society Special Group in Health Psychology Annual Conference, Southampton, UK. 1997.

[43] Erdfelder E, FAul F, Buchner A, Lang AG. Statistical power analyses using G*Power 3.1: Tests for correlation and regression analyses. Behav Res Methods. 2009;41(4):1149–60. doi: 10.3758/BRM.41.4.1149 19897823

[44] IBM Corp. IBM SPSS Statistics for Windows, Version 26.0. 2019. 2019.

[45] RStudio T. RStudio: Integrated Development for R. RStudio [Internet]. Boston, MA; 2020. Available from: https://www.rstudio.com/.

[46] Kim H-Y. Statistical notes for clinical researchers: assessing normal distribution (2) using skewness and kurtosis. Restor Dent Endod. 2013;38(1):52. doi: 10.5395/rde.2013.38.1.52 23495371 PMC3591587

[47] Krippendorff K. The content analysis reader. California: Sage; 2009.

[48] Braun V, Clarke V. Using thematic analysis in psychology. Qual Res Psychol. 2006;3(2):77–101.

[49] Smith BW, Epstein EM, Ortiz JA, Christopher PJ, Tooley EM. The Foundations of Resilience: What Are the Critical Resources for Bouncing Back from Stress? In 2013.

[50] Whitlock E. Kilburn J. Distraction teckniques and alternative coping strategies [Internet]. Cornell research program on self-injurious behavior in adolescents and young adults. 2012 [cited 2019 Dec 11]. Available from: http://www.selfinjury.bctr.cornell.edu/documents/distraction-tech-and-alts.pdf.

[51] Folkman S. Positive psychological states and coping with severe stress. Soc Sci Med. 1997;45(8):1207–21. doi: 10.1016/s0277-9536(97)00040-3 9381234

[52] Ryan S. Cole KR. From advocate to activist? Mapping the experiences of mothers of children on the autism spectrum. J Appl Res Intellect Disabil. 2009;22(1):43–53.

[53] Seymour M, Wood C, Giallo R, Jellett R. Fatigue, stress and coping in mothers of children with an autism spectrum disorder. J Autism Dev Disord. 2013 Jul;43(7):1547–54. doi: 10.1007/s10803-012-1701-y 23124359

[54] Gray DE. Coping over time: The parents of children with autism. J Intellect Disabil Res. 2006 Dec;50(12):970–6. doi: 10.1111/j.1365-2788.2006.00933.x 17100957

[55] Lazarus R. S., & Folkman S. Coping and adaptation. In: The handbook of behavioral medicine,. 1984. p. 282–325.

[56] Folkman S, Lazarus RS. If It Changes It Must Be a Process: Study of Emotion and Coping During Three Stages of a College Examination. Vol. 48. 1985. 10.1037//0022-3514.48.1.150 2980281

[57] Coyne J. C. Tennen H. Positive psychology in cancer care: Bad science, exaggerated claims, and unproven medicine. Ann Behav Med. 2010;39(1):16–26. doi: 10.1007/s12160-009-9154-z 20146038 PMC2858800

[58] Sin NL, Lyubomirsky S. Enhancing well-being and alleviating depressive symptoms with positive psychology interventions: A practice-friendly meta-analysis. J Clin Psychol. 2009;65(5):467–87. doi: 10.1002/jclp.20593 19301241

[59] Phelps C, Bennett P, Iredale R, Anstey S, Gray J. The development of a distraction-based coping intervention for women waiting for genetic risk information: A phase 1 qualitative study. Psychooncology. 2006;15(2):169–73. doi: 10.1002/pon.937 15929031

[60] Lai WW, Goh TJ, Oei TPS, Sung M. Coping and Well-Being in Parents of Children with Autism Spectrum Disorders (ASD). J Autism Dev Disord. 2015 Aug 27;45(8):2582–93. doi: 10.1007/s10803-015-2430-9 25800867

